# Re-interpreting the data on the cost and effectiveness of population screening for colorectal cancer in Australia

**DOI:** 10.1186/1743-8462-2-10

**Published:** 2005-05-18

**Authors:** Nicholas Graves, Loretta McKinnon, Barbara Leggett, Beth Newman

**Affiliations:** 1 School of Public Health, Queensland University of Technology, Victoria Park Road, Kelvin Grove QLD, 4059, Australia; 2 Queensland Institute for Medical Research, 300 Herston Road, Herston, QLD 4006, Australia

## Abstract

Three studies report estimates of the cost and effectiveness of alternate strategies for screening the average-risk Australian population for colorectal cancer. The options considered are faecal occult blood testing, double contrast barium enema, sigmoidoscopy and colonoscopy. At present, there is no consensus over which screening method is optimal by the economic criterion. Also, the existing studies report a mixture of average and incremental cost-effectiveness ratios derived from data collected between 1994 and 2002. We suggest *average *cost-effectiveness ratios are not useful for decision-making and illustrate how they differ from the preferred *incremental *cost-effectiveness ratio. We then update the cost data reported in the three studies to 2002 prices and calculate incremental cost-effectiveness ratios where not previously available. Our re-analysis of one study contradicts the conclusions drawn by the authors, who had only calculated average cost-effectiveness ratios. In particular, we find their recommendation of population screening with colonoscopy would cause, annually, between 33 and 1,322 years of life to be lost and between $M17 and $M87 to be wasted. Based on updated cost data and the incremental analysis, our findings indicate that population screening using biennial faecal occult blood testing ($39,459 per life-year gained), annual faecal occult blood testing ($30,556 per life-year gained) and colonoscopy ($26,587 per life-year gained) are cost-effective. Hence, the decision over which method of screening is optimal remains ambiguous across the three studies. We recommend policy-makers choose the study they believe produces the most accurate estimates of cost and health effect, identify their willingness to pay for health benefits and consider other issues relevant to the decision.

## Introduction

In 1996, Salkeld et al. [1] found that screening the average-risk Australian population for colorectal cancer using a faecal occult blood test (FOBT), compared to existing practice, would cost $24,660 per life-year gained (LYG). Due to uncertainty regarding the effectiveness of FOBT screening, they report a range of values, between $12,695 and $67,848 per LYG. Randomised controlled trials of population screening with FOBT conducted in the UK [2] and Denmark [3], but published after Salkeld et al.'s study, have reduced this uncertainty. The cost-effectiveness analyses based on the UK trial data [4] suggest a cost per life-year gained between £1,371–£5,685 (approximately $AU3,370–13,974) and the analysis of the Danish trial data [5] suggest a cost per life-year gained between 17,000–42,000DKK (approximately $AU3,916–9,672).

Three years before publication of Salkeld's study Bolin [6] discussed the advantages of using colonoscopy for population screening, and in 1996, he suggested colonoscopy was cost-effective [7]. In 1997, he asked whether the time had come to use colonoscopy for population screening in Australia [8]. Kermond [9] responded suggesting double contrast barium enema (DCBE) should not be overlooked arguing colonoscopy is 10 times more expensive, false negatives still occur and complication rates are higher. Bolin argued, in the same issue of the MJA, that the sensitivity of colonoscopy exceeds DCBE, the complication rate is only 0.1% and cost differentials are actually less than those suggested by Kermond [9].

Bolin also claimed that FOBT at one and three years and colonoscopy at 10 years, assuming a 10-year period during which time the cancer is detectable and curable (known as the dwell time), are cost-effective modes of CRC screening, probably referring to data subsequently published in 1999 [10]. For this research, the authors substituted Australian values for cost parameters into a US model of the cost-effectiveness of CRC screening [11] and generalised the results to the Australian population. They reported change in cost and change in life-years gained, as compared to existing practice, for competing screening strategies that encompass FOBT, colonoscopy, flexible sigmoidoscopy and DCBE [10]. By assuming that society is willing to pay up to $US40,000 (approximately $AU65,449 in 2002 prices) per LYG, Bolin proposed that annual FOBT, triennial FOBT, triennial DCBE, five-yearly DCBE, five-yearly colonoscopy and ten-yearly colonoscopy are all cost-effective and concluded that physicians have the option of offering individuals a range of screening alternatives, including colonoscopy [10]. Since publishing the research Bolin has argued, on four separate occasions that population screening with colonoscopy is cost-effective [12-15]. The last of these, in 2002 [14], provoked Macrae and Hebbard [16] to criticise Bolin's interpretation of epidemiological data.

In 2004, O'Leary et al. [17] also addressed the economic questions around population screening in Australia. They estimated the cost-effectiveness, compared to existing practice, of FOBT, flexible sigmoidoscopy and colonoscopy, and found flexible sigmoidoscopy and colonoscopy were cost-effective but FOBT was not.

There are important differences in the way that Salkeld et al. [1], Bolin et al. [10] and O'Leary et al. [17] report the Australian cost and effectiveness data. Salkeld et al. reports an incremental cost-effectiveness ratio, O'Leary et al. reports both average and incremental cost-effectiveness ratios, however they draw their conclusions from an average analysis. Bolin only calculates average cost-effectiveness ratios. The correct ratio for decision-making is an incremental cost-effectiveness ratio: McMahon [18] argues the use of average ratios is not meaningful; Drummond [19] and Gold [20] both discuss why incremental rather than average cost-effectiveness ratios are relevant for decision making; and, both Torgerson [21] and Neuhauser & Lewicki [22] provide examples of how average analyses muddy the waters. In their much-cited 1975 paper, Neuhauser & Lewicki [22] reviewed data on screening for CRC. They illustrated that repeatedly testing a stool sample up to six times, when a previous test result was negative, would capture all cases of CRC, at an average cost per case of $2451. They also did an incremental analysis, with the same data, and showed the incremental cost per case detected, from the fifth to sixth round of testing was $47 million. This illustrates that average analyses can be grossly misleading.

We have four objectives in this paper: first, to demonstrate why incremental, not average, cost-effectiveness ratios should be used for decision-making; second, to update the cost data reported by Salkeld et al. [1], Bolin et al. [10] and O'Leary et al. [17] to 2002 Australian dollar prices; third, to calculate incremental cost-effectiveness ratios from the Bolin data; and fourth, to discuss the results of our re-analysis, comparing the outcomes from the three previous studies. This will provide readers with an up-to-date and appropriate assessment of the existing cost-effectiveness data for population-based CRC screening programmes in Australia.

## Defining average & incremental cost-effectiveness ratios

Average cost-effectiveness ratios for health care interventions are the amounts by which costs change from a baseline comparator (Δ*C*) divided by the amount by which health benefits change from a baseline comparator (Δ*E*). The baseline comparator is often existing practice. We illustrate this method with hypothetical data in Table 1 by presenting the change in costs and health effects that arise from four competing health care alternatives. If we remain with Existing Practice, there is no change in cost or health effect. However, if we are committed to generating health effects and we wish to be efficient, then we should choose the cheapest option that improves health outcomes. The data in Table 1 show that Intervention 4 generates the best ratio of (Δ*C*) and (Δ*E*) when compared to Existing Practice. Cost changes by $145,000 and health effects change by 150 and the cost per LYG is $967.

**Table 1:** An illustration of average cost-effectiveness ratios for four competing hypothetical health care interventions

	Change in cost ($)	Change in health effect (Life-years Gained)	Average cost-effectiveness ($)
	(Δ*C*)	(Δ*E*)	(Δ*C*) divided by (Δ*E*)

Existing Practice	0	0	
Intervention 1	200,000	12	16,667
Intervention 2	75,000	15	5,000
Intervention 3	300,000	250	1,200
Intervention 4	145,000	150	967

**Figure 1:**
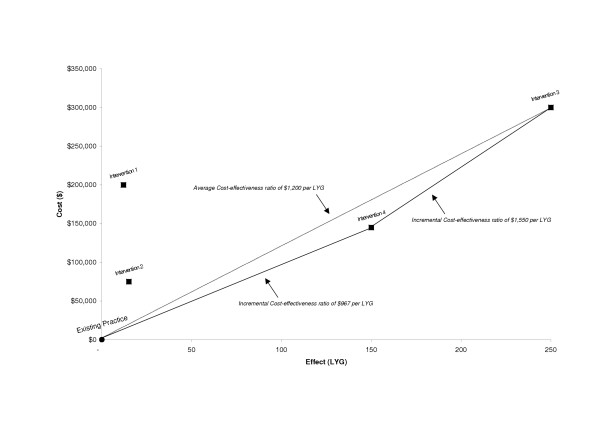
Change in cost and change in effect from four hypothetical health care interventions.

We reject Interventions 1 and 2. The reasons lie in Figure 1 (which is a graph of the data in Table 1). Intervention 1 generates less health benefit and higher costs than Intervention 4, a situation described as 'simple dominance'. For this comparison, Intervention 4 is preferred on both costs and outcomes. Intervention 2 also generates less health effect but differs from Intervention 1 in that it's cheaper than Intervention 4; however, the cost per LYG from Intervention 2 is greater than the cost per LYG from Intervention 4. This situation is known as 'extended dominance' and is only relevant if the cost of Intervention 4 ($145,000) exceeds the total amount of money available to the decision-maker. Rather than choosing Intervention 2 over Intervention 4, it would be better (more productively efficient) to choose some blend of existing practice and Intervention 4. This implies that some proportion of the population would receive Intervention 4 and the remainder would receive existing practice. This raises questions of equity of access and so poses another set of problems for decision-makers. If the available budget exceeds $145,000, there is a further question to consider. Do we invest in the more costly but more effective Intervention 3? Some care is required when making this decision. The average cost-effectiveness ratio for Intervention 3 ($1,200 per LYG), represented by the dashed line on Figure 1, is misleading. It's calculated by comparing Intervention 3 to existing practice; yet, the relevant decision is whether we should invest in Intervention 3 given that we have established Intervention 4 as the most cost-effective option. We must consider the incremental changes in cost and health effects compared to the next best alternative, Intervention 4. The incremental cost-effectiveness ratios for Interventions 4 and 3 are marked with solid lines on Figure 1. When a more effective alternative also costs more, then the decision-maker must compare the increased cost with the increased effects [19]. The only way to achieve this is to conduct an incremental analysis, which we illustrate in Table 2. Investing in Intervention 3, as compared to 4, changes total costs by $155,000 and LYG by 100, yielding an incremental cost-effectiveness ratio of $1,550 per LYG not the $1,200 per LYG as previously estimated using average cost-effectiveness ratios and illustrated in Table 1.

**Table 2:** An illustration of average and incremental cost-effectiveness ratios for the two remaining hypothetical health care interventions

Intervention	Cost ($)	Incremental changes in cost ($)	Effectiveness (LYG)	Incremental changes in effectiveness (LYG)	Average cost-effectiveness ratio ($)	Incremental cost-effectiveness ratio ($)
	**(a)**	**(b)**	**(c)**	**(d)**	**(a)/(c)**	**(b)/(d)**

Existing Practice	0		0			
Intervention 4	145,000	145,000	150	150	967	967
Intervention 3	300,000	155,000	250	100	1,200	1,550

Note: Interventions 1 and 2 have been rejected on the grounds of 'simple' and 'extended' dominance, see text for a discussion

Imagine you have decided to take a holiday in a beach resort. You face a decision between a standard apartment for $1,000 and a Penthouse apartment for $1,300. Because you have decided to take the holiday (and so one of the apartments), it is the difference in cost ($300) that you compare to the difference in benefit (penthouse vs. standard apartment). If you don't perceive the additional benefit to be worth the extra $300, then you reject the penthouse. This simple example illustrates the importance of thinking about decisions in terms of incremental changes. An average analysis with both options compared to 'no holiday' may lead to a bad decision.

## Methods for re-analysis and the results

We converted Bolin's $US dollar estimates to Australian dollars with the exchange rate reported in the original article [10] and adjusted the estimates reported by Bolin, Salkeld and O'Leary to 2002 prices using a health price index [23]. As Salkeld and O'Leary reported incremental cost-effectiveness ratios, we only need to calculate incremental cost-effectiveness ratios from the Bolin data. We achieved this by inputting the reported estimates of (Δ*C*) and (Δ*E*) into decision analysis software [24]. Assuming a 5-year dwell time (the period during which cancer can be detected and cured), we include 13 screening strategies and the existing practice comparator. For a 10-year dwell time, estimates of (Δ*C*) and (Δ*E*)were reported for different, additional, frequencies of FOBT, flexible sigmoidoscopy and DCBE screening, resulting in 27 strategies and the existing practice comparator. We ranked all strategies by increasing cost, estimated incremental cost and effectiveness, and excluded all strategies for which other options prevailed on the basis of 'simple' or 'extended' dominance. Finally, we reported the strategies not excluded due to either 'simple' or 'extended' dominance and present the relevant incremental cost-effectiveness ratios.

In Table 3, we describe all strategies evaluated by the authors of the three studies.

**Table 3:** Descriptions of the screening strategies included in the re-analysis

Screening strategy	Description of screening strategy	Salkeld [1]	Bolin [10] (5-year dwell time)	Bolin [10] (10-year dwell time)	O'Leary [17]
Existing practice	Existing screening practices		X	X	X
COL10	10-yearly colonoscopy		X	X	X
COL5	5-yearly colonoscopy		X	X	
COL	one off screening colonoscopy at age 50		X	X	
DCBE	one off double contrast barium enema			X	
DCBE10	10-yearly double contrast barium enema			X	
DCBE15	15-yearly double contrast barium enema			X	
DCBE20	20-yearly double contrast barium enema			X	
DCBE3	3-yearly double contrast barium enema		X	X	
DCBE5	5-yearly double contrast barium enema		X	X	
FOBT10	10-yearly faecal occult blood test			X	
FOBT15	15-yearly faecal occult blood test			X	
FOBT2	2-yearly faecal occult blood test			X	X
FOBT20	20-yearly faecal occult blood test			X	
FOBT5	5-yearly faecal occult blood test			X	
FOBT	one off faecal occult blood test			X	
FOBT1	annual faecal occult blood test	X	X	X	X
FOBT1+DCBE3	annual faecal occult blood test and 3-yearly double contrast barium enema		X	X	
FOBT1+DCBE5	annual faecal occult blood test and 5-yearly double contrast barium enema		X	X	
FOBT1+FSIG3	annual faecal occult blood test and 3-yearly flexible sigmoidoscopy		X	X	
FOBT1+FSIG5	annual faecal occult blood test and 5-yearly flexible sigmoidoscopy		X	X	
FOBT3	3-yearly faecal occult blood test		X	X	
FSIG	flexible sigmoidoscopy once only			X	
FSIG10	10-yearly flexible sigmoidoscopy			X	X
FSIG15	15-yearly flexible sigmoidoscopy			X	
FSIG20	20-yearly flexible sigmoidoscopy			X	
FSIG3	3-yearly flexible sigmoidoscopy		X	X	
FSIG5	5-yearly flexible sigmoidoscopy		X	X	

**Figure 2:**
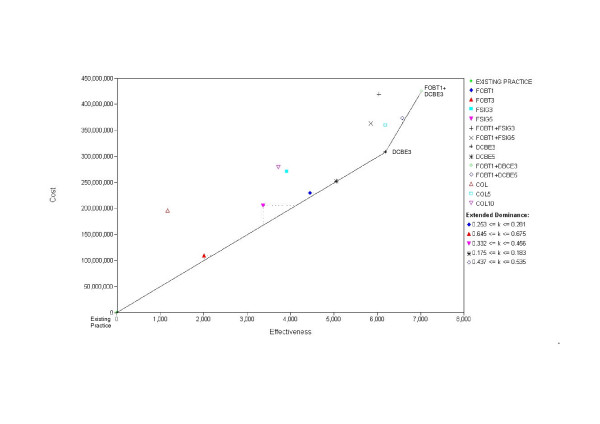
Updated costs and effects for Bolin et al's 13 tested strategies, assuming a five-year dwell time.

In Figure 2, we illustrate the costs and effects of all strategies reported by Bolin, assuming a 5-year dwell time. The strategies to the left and above the cost-effective frontier, defined by the solid line, are excluded by either 'simple' or 'extended' dominance. The three remaining (un-dominated) strategies, that define the cost-effective frontier, are EXISTING PRACTICE, DCBE3 and FOBT1+DBCE3. In Table 4, we report all corresponding costs, health benefits and cost-effectiveness ratios, and indicate the options that are 'simply' dominated. In Table 5, we report the incremental cost-effectiveness ratios for the options that survive the tests of 'simple' or 'extended' dominance; these options define the cost-effective frontier.

**Table 4:** Estimates of costs in 2002 prices, health benefits and cost-effectiveness from Bolin *et al*. [10], assuming a five-year dwell time

Strategy	Cost ($)	Incremental changes in cost ($)	Effectiveness (LYG)	Incremental changes in effectiveness (LYG)	Average cost-effectiveness ratio ($)	Incremental cost-effectiveness ratio ($)
	**(a)**	**(b)**	**(c)**	**(d)**	**(a)/(c)**	**(b)/(d)**

EXISTING PRACTICE	0		0		(Undefined)	
FOBT3	109,167,314	109,167,314	2,010	2,010	54,312	54,312
COL	195,987,062	86,819,748	1,166	-844	168,085	(Simply Dominated)
FSIG5	205,570,434	96,403,120	3,365	1,355	61,091	71,146
FOBT1	230,156,355	24,585,921	4,447	1,082	51,755	22,723
DCBE5	253,881,621	23,725,266	5,050	603	50,274	39,345
FSIG3	271,052,169	17,170,548	3,909	-1,141	69,341	(Simply Dominated)
COL10	278,701,522	24,819,901	3,718	-1,332	74,960	(Simply Dominated)
DCBE3	307,911,416	54,029,795	6,184	1,134	49,792	47,645
COL5	360,264,079	52,352,663	6,181	-3	58,286	(Simply Dominated)
FOBT1+FSIG5	364,595,167	56,683,751	5,849	-335	62,335	(Simply Dominated)
FOBT1+DCBE5	373,803,843	65,892,427	6,573	389	56,870	169,389
FOBT1+FSIG3	420,786,416	46,982,573	6,032	-541	69,759	(Simply Dominated)
FOBT1+DBCE3	424,911,339	51,107,496	7,020	447	60,529	114,334

**Table 5:** Incremental cost-effectiveness ratios in 2002 prices for the preferred (not dominated) strategies from Bolin et al. [10], assuming a five-year dwell time

Strategy	Cost ($)	Incremental changes in cost ($)	Effectiveness (LYG)	Incremental changes in effectiveness (LYG)	Average cost-effectiveness ratio ($)	Incremental cost-effectiveness ratio ($)
	**(a)**	**(b)**	**(c)**	**(d)**	**(a)/(c)**	**(b)/(d)**

EXISTING PRACTICE	0		0		(Undefined)	
DCBE3	307,911,416	307,911,416	6,184	6,184	49,792	49,792
FOBT1+DBCE3	424,911,339	116,999,923	7,020	836	60,529	139,952

Note: Other options rejected on the grounds of 'simple' and 'extended' dominance, see text for a discussion.

**Figure 3:**
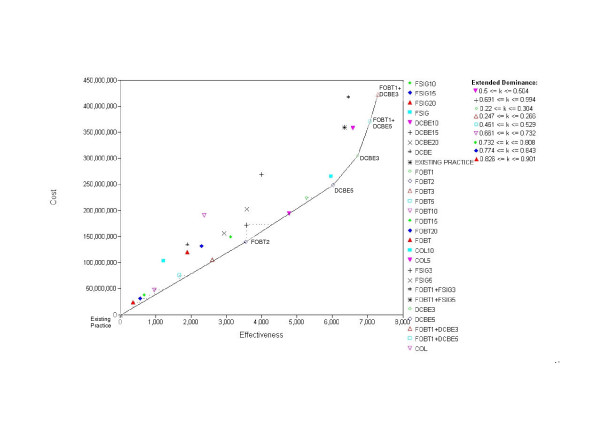
Updated costs and effects for Bolin et al's 27 tested strategies, assuming a ten-year dwell time.

In Figure 3, we illustrate the costs and effects for all strategies reported by Bolin, assuming a 10-year dwell time. In this case EXISTING PRACTICE, FOBT2, DCBE5, DCBE3, FOBT1+DCBE5 and FOBT1+DCBE3 define the cost-effective frontier. In Table 6, we report all corresponding costs, health benefits and cost-effectiveness ratios, and indicate the options that are 'simply' dominated. In Table 7, we report the incremental cost-effectiveness ratios for the options that survive the tests of 'simple' or 'extended' dominance; again, these options define the cost-effective frontier.

**Table 6:** Estimates of costs in 2002 prices, health benefits and cost-effectiveness from Bolin *et al*. [10], assuming a 10-year dwell time

Strategy	Cost ($)	Incremental changes in cost ($)	Effectiveness (LYG)	Incremental changes in effectiveness (LYG)	Average cost-effectiveness ratio ($)	Incremental cost-effectiveness ratio ($)
	**(a)**	**(b)**	**(c)**	**(d)**	**(a)/(c)**	**(b)/(d)**

EXISTING PRACTICE	0		0		(Undefined)	
FOBT	24,429,089	24,429,089	352	352	69,401	69,401
FOBT20	31,633,953	7,204,864	558	206	56,692	34,975
FOBT15	37,620,230	5,986,277	681	123	55,243	48,669
FOBT10	47,472,852	9,852,622	951	270	49,919	36,491
FOBT5	75,561,379	28,088,527	1,674	723	45,138	38,850
FSIG	102,900,905	27,339,526	1,222	-452	84,207	(Simply Dominated)
FOBT3	105,521,804	29,960,425	2,605	931	40,507	32,181
FSIG20	120,362,301	14,840,497	1,892	-713	63,616	(Simply Dominated)
FSIG15	131,780,307	26,258,503	2,294	-311	57,446	(Simply Dominated)
DCBE	134,697,657	29,175,853	1,896	-709	71,043	(Simply Dominated)
FOBT2	140,117,791	34,595,987	3,551	946	39,459	36,571
FSIG10	148,850,170	8,732,379	3,127	-424	47,602	(Simply Dominated)
DCBE20	158,390,199	18,272,408	2,939	-612	53,893	(Simply Dominated)
DCBE15	173,448,383	33,330,592	3,566	15	48,639	2,222,039
COL	190,862,405	17,414,022	2,368	-1,198	80,601	(Simply Dominated)
DCBE10	194,089,298	20,640,915	4,778	1,212	40,621	17,030
FSIG5	204,328,539	10,239,241	3,583	-1,195	57,027	(Simply Dominated)
FOBT1	224,367,390	30,278,092	5,271	493	42,566	61,416
DCBE5	248,112,291	23,744,901	6,023	752	41,194	31,576
COL10	265,297,565	17,185,274	5,970	-53	44,438	(Simply Dominated)
FSIG3	270,484,399	22,372,108	3,993	-2,030	67,740	(Simply Dominated)
DCBE3	304,301,903	56,189,612	6,720	697	45,283	80,616
COL5	357,822,831	53,520,928	6,583	-137	54,356	(Simply Dominated)
FOBT1+FSIG5	361,026,560	56,724,657	6,344	-376	56,908	(Simply Dominated)
FOBT1+DCBE5	370,181,240	65,879,337	7,076	356	52,315	185,054
FOBT1+FSIG3	417,677,588	47,496,348	6,457	-619	64,686	(Simply Dominated)
FOBT1+DCBE3	422,777,702	52,596,462	7,299	223	57,923	235,859

**Table 7:** Incremental cost-effectiveness ratios in 2002 prices for the preferred (not dominated) strategies from Bolin et al. [10], assuming a 10-year dwell time

Strategy	Cost ($)	Incremental changes in cost ($)	Effectiveness (LYG)	Incremental changes in effectiveness (LYG)	Average cost-effectiveness ratio ($)	Incremental cost-effectiveness ratio ($)
	**(a)**	**(b)**	**(c)**	**(d)**	**(a)/(c)**	**(b)/(d)**

EXISTING PRACTICE	0		0		(Undefined)	
FOBT2	140,117,791	140,117,791	3,551	3,551	39,459	39,459
DCBE5	248,112,291	107,994,500	6,023	2,472	41,194	43,687
DCBE3	304,301,903	56,189,612	6,720	697	45,283	80,616
FOBT1+DCBE5	370,181,240	65,879,337	7,076	356	52,315	185,054
FOBT1+DCBE3	422,777,702	52,596,462	7,299	223	57,923	235,859

Note: Other options rejected on the grounds of 'simple' and 'extended' dominance, see text for a discussion

The incremental cost-effectiveness ratio derived from the Salkeld data, in 2002 prices, for FOBT, is $30,556 per LYG, and the 2002 incremental cost-effectiveness ratios from the O'Leary data are $17,356 per LYG for FSIG and $26,587 per LYG for COL.

These results define the screening options that are preferred (ie, not dominated), by the measure of cost-effectiveness, for population-based CRC screening in Australia. However, the decision over which to choose depends on additional factors that we discuss next.

## Discussion

We defined average and incremental cost-effectiveness ratios and emphasise the latter are relevant for decision-making. We calculated incremental cost-effectiveness ratios, in 2002 prices, for a number of population screening strategies, for which Bolin had previously reported average cost-effectiveness ratios. For Bolin's estimates of (Δ*C*) and (Δ*E*), for a 5-year dwell time, we found only DCBE3 ($49,792 per LYG) and FOBT1+DCBE3 ($139,952 per LYG) were preferred (not dominated). For a 10-year dwell time, FOBT2 ($39,459 per LYG), DCBE5 ($43,687 per LYG), DCBE3 ($80,616 per LYG), FOBT1+DCBE5 ($185,054 per LYG) and FOBT1+DCBE3 ($235,859 per LYG) were preferred (not dominated).

Incremental cost-effectiveness ratios are useful for decision-making when a ceiling value for a LYG is specified. Bolin argued in 1996 [7] that $US40,000 per LYG was the relevant cut-off (approximately $AU65,449 in 2002 prices). We prefer a decision rule described by Garber & Phelps [25] that states a LYG is worth approximately twice the median annual per capita income. They derived this value from a model of optimal lifetime spending for medical care and explored its relationship to the cost-effectiveness criterion. They evaluated the model in terms of maximizing utility for individuals, with utility a function of income and health. Their rule implies, for Australia, a rational cut-off for one LYG is approximately $AU39,000 [26].

If we apply this rule to our interpretation of Bolin's data, we wouldn't recommend any additional population screening activities for the 5-year dwell time, and for a 10-year dwell time, we would only recommend FOBT2. Based on the O'Leary and Salkeld data we recommend COL and FOBT1, respectively. The results in Tables 4 and 6 illustrate the colonoscopy strategies, championed by Bolin, would cause between 33 and 1,322 years of life to be lost and between $M17 and $M87 to be wasted. We showed that all colonoscopy options were dominated by more cost-effective alternatives.

Despite our re-analysis, the decision over which model of CRC screening is optimal for the Australian, average-risk population remains ambiguous. While the incremental cost-effectiveness ratios from two studies support annual FOBT [1] or biennial FOBT [10], the most recent study supports colonoscopy [17]. At least we now have incremental cost-effectiveness ratios in 2002 prices. While two of the studies agree that FOBT screening is preferred, we have not investigated why the O'Leary [17] analysis leads to a different conclusion. The answer may be sought in a careful assessment of model structures, the particular perspectives adopted for each analysis and the values used for the parameters, which is beyond the scope of this commentary. In addition, we haven't attempted to model the effect of uncertainty on the conclusions. This would require access to the models, data and software used in each of the previous three studies. Policy-makers should review the three papers and make a judgement over which they believe produces the best estimates of change in cost and health benefit, identify their willingness to pay for the proposed health benefits and make their decision in the context of other logistic, social and political issues.

## Competing interests

The author(s) declare that they have no competing interests.
